# Beyond Disaster Preparedness: Building a Resilience-Oriented Workforce for the Future

**DOI:** 10.3390/ijerph14121563

**Published:** 2017-12-13

**Authors:** Jaime Madrigano, Anita Chandra, Tracy Costigan, Joie D. Acosta

**Affiliations:** 1 RAND Corporation, 1200 South Hayes Street, Arlington, VA 22202, USA; chandra@rand.org (A.C.); jacosta@rand.org (J.D.A.); 2 Robert Wood Johnson Foundation, 50 College Road East, Princeton, NJ 08540, USA; tcostigan@rwjf.org

**Keywords:** resilience, workforce, disaster preparedness

## Abstract

Enhancing citizens’ and communities’ resilience is critical to adapt successfully to ongoing challenges faced by communities, as well as acute shocks resulting from disasters. While significant progress has been made in this area, several research and practice gaps remain. A crucial next step to advance resilience is the development of a resilience-oriented workforce. This narrative review examines existing literature to determine key components of a resilience-oriented workforce, with a focus on organizational structures, training and education, and leadership models. Reviewed articles spanned a variety of study types, including needs assessments of existing workforce, program evaluations, and reviews/commentaries. A resilience-oriented workforce spans many disciplines and training programs will need to reflect that. It requires a collaborative organizational model that promotes information sharing structures. Leadership models should foster a balance between workforce autonomy and operation as a collective entity. Optimal strategies to develop a resilience-oriented workforce have yet to be realized and future research will need to collect and synthesize data to promote and evaluate the growth of this field.

## 1. Introduction

In recent years, the array of social and physical stresses that people and communities experience has multiplied [1]. Official declarations of natural, manmade, and technological disasters in the United States (and globally) have increased in the past decade, and these types of disasters overlay the day-to-day challenges that many communities already confront, including economic difficulties, structural racism, and environmental stress [2]. In 2016 alone, the United States sustained fifteen separate weather disasters each causing damage amounting to a billion dollars or more [3] and resulting in significant loss of life, as well as other economic and societal impacts to the affected areas. In 2017, we have observed the devastations of disasters in Mexico, the Caribbean, India, and the United States. In addition to natural, manmade, and technological disasters, other acute shocks, such as active shooter events, have also increased in frequency over the last decade [4]. 

Government agencies and policymakers have called for enhancing citizens’ and communities’ resilience to prepare populations in advance of disasters with an emphasis on promoting individual and community resilience through scholarly, policy, and programmatic efforts [5]. Resilience can be defined as the capacity of a community to recover from disasters and from other problems, such as violence and economic downturns, and emerge stronger and better able to withstand future adverse events [6,7,8]. Much of the prior resilience research has been conducted in the context of disasters and other traumatic events. This work has yielded some consensus around the factors that make a resilience approach unique from traditional emergency preparedness. Namely, resilience is more broadly defined, focuses on the whole community, is relationship based (vs. plan-based), and places relatively more emphasis on population strengths (vs. vulnerabilities), organizational assets (resources, money, skills, relationships), and sustainable development. Also, while preparedness has typically been phased (preparedness-response-recovery) or episodic, resilience as previously defined is an ongoing capacity building [6,8,9,10]. While significant progress has been made to advance this type of resilience approach, including multiple initiatives led by government agencies and philanthropic organizations to implement resilience frameworks [11,12,13], there remain several research and practice gaps that have prevented the full optimization of resilience.

The next phase to advance resilience will require greater focus on the factors that contribute to field building, and within that, the development of a resilience-oriented workforce. For this paper, we define a resilience-oriented workforce not as a single and unique set of professionals trained in resilience, but rather a goal state whereby all professions involved in protecting and promoting health of places and people possess the capacity (knowledge, attitudes, and skills) necessary to be integrated with each other (not just connected) and thus resilient in the face of a disaster or other widespread stress. Prior research has suggested that a resilient community requires strong connections between neighborhoods and community organizations, and between a diversity of local and non-governmental groups [6,7,8]. These professions necessarily include those focused on building and protecting places (e.g., engineers, urban planners, architects) and those focused on growing healthy people (e.g., health care providers, emergency managers, social service providers, faith-based organizations, public health practitioners, law enforcement).

Given the broad scope of resilience and the diversity of professions involved, this paper takes a resilience field building perspective to workforce development—aiming to connect fragmented professions around the challenge of creating resilient communities [14]. To ensure that a field builds, deepens, and has meaningful impact, several areas must be addressed; namely, creating shared identity, deepening research evidence, instituting structures for collaboration, activating research and practice constituencies that can mobilize, developing effective leaders, and pursuing integrative work as the priority, many of which are often contained in workforce development. Fields like resilience require structures for meaningful collaboration and opportunities for effective leadership to align disciplines and sectors for joint action [15,16,17]. As noted in other fields (e.g., teacher education), it is difficult to progress a field like resilience without leadership and broader workforce development. Resilience necessitates new ways of thinking (e.g., new ways of putting risks and assets together for planning), and new ways of doing (e.g., new ways of working together to problem-solve), to advance resilience-based research and policy [18]. Building a capable workforce is the foundation for making these changes.

This paper arose out of discussions at the Resilience Roundtable [8], a meeting of approximately 80 leading researchers, practitioners, and policymakers convened by the Robert Wood Johnson Foundation and RAND Corporation in June 2016. During the Roundtable discussions, participants underscored many of the challenges confronting the development of a robust workforce that can address the need for more transdisciplinary and integrated action. That is to say, actors from different disciplines need to work jointly to create new conceptual, methodological, and translational innovations that integrate and move beyond discipline-specific approaches to address a common problem [19]. Drawing upon these discussions, we set out to understand the key components that could drive the development of a resilience-oriented workforce. Previous efforts to advance workforce development (e.g., within the field of public health) have demonstrated a need for organizational structures and skill building [20,21], and this is particularly relevant for the field of resilience due to the myriad of disciplines involved and the emphasis on both individual and community organizing. Given this background, we aimed to answer three questions: (1) What organizational structures will support connections needed for a resilience-oriented workforce? (2) What elements of training will support resilience-oriented education? and (3) What are leadership models with a resilience orientation? This narrative review summarizes the existing literature on resilience-oriented leadership or workforce development as it relates to these three research questions to synthesize current approaches and ideas in the field and determine areas of opportunity going forward. 

## 2. Materials and Methods

**Figure 1:**
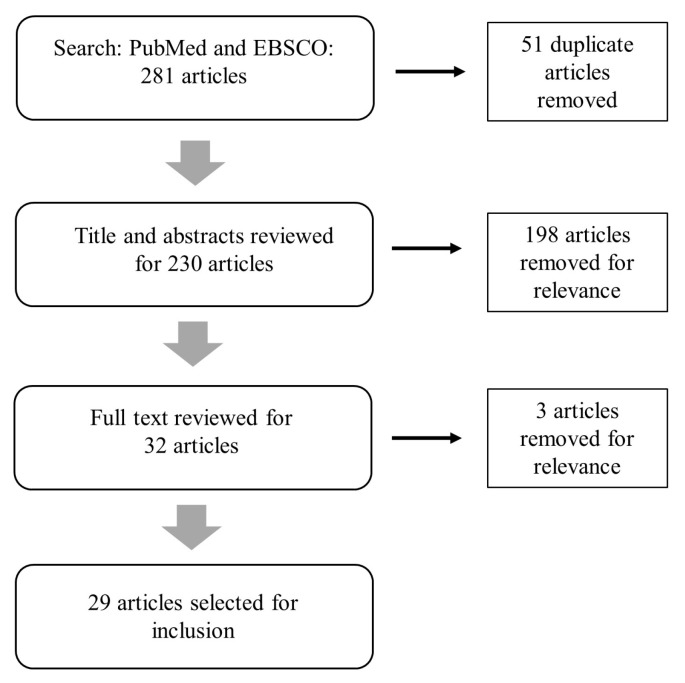
Literature search process.

We performed a search of the peer-reviewed literature using both Medline (PubMed) and Academic Search Complete (EBSCO) databases. Articles from 2000 or later were identified using a title and abstract search. The following terms were used to perform the search: workforce AND (resilienc* OR “disaster preparedness”). Many lessons learned about what it takes to support whole communities during stressful times are from the field of disaster preparedness, so we included both the term resilienc* and “disaster preparedness” in our search. The literature search identified a total of 281 articles. After removal of duplicate articles, 230 remained. A title and abstract review revealed that the vast majority of articles either captured how to make individual leaders more resilient to change and adversity or how to deal with burnout or compassion fatigue issues among disaster response and recovery workers. While these are important drivers of effective leadership and are key to promoting a *resilient workforce*, they do not help us answer the motivating questions about how to build a *resilience-oriented workforce* and, therefore, were eliminated from further review. With this elimination, a total of 32 articles were available for full text review. Three articles were excluded because they did not have a specific focus on workforce issues, and 29 articles were selected for final inclusion (Figure 1).

Final article selection was motivated by our three research questions. The final set of articles was reviewed and catalogued with the use of a data abstraction form (DAF). The DAF facilitated systematic evaluation by capturing from each document several elements regarding content (e.g., type of study and summary of key findings). The DAF was used to catalogue the specific segment or segments of the workforce that were under focus, whether the study was for a specific preparedness strategy (e.g., disaster preparedness) or resilience, more broadly, and which research question it addressed. 

## 3. Resilience-Oriented Workforce Studies

The majority (*n* = 20) of articles selected for full review pertained to disaster preparedness for health care professionals (including physicians, nurses, and allied health professionals) (Table 1). Nurses were the most common workforce segment featured within the healthcare professional literature. A smaller set of articles examined workforce for disaster preparedness, but not specifically for healthcare professionals (*n* = 5), or workforce within the context of resilience, more broadly, but not specifically related to disaster preparedness (*n* = 4). The articles spanned a variety of study types, including needs assessments of existing workforce, program evaluations, and reviews/commentaries. Findings from our review are summarized below by the three previously identified research questions.

### 3.1. What Organizational Structures Will Support the Connections Needed for a Resilience-Oriented Workforce?

To support the connections needed for a resilience-oriented workforce, it is necessary to have structures in place that allow sharing of information between the people- and place-focused professions, and across multiple levels and branches of government, and the private sector [22,23]. Example structures may include coalitions, public-private partnerships, integrated data systems, multi-sector planning bodies, and community advisory boards. A clear need for such structures was demonstrated in the response to the 11 September terrorist attack on the World Trade Center, where problems arose when organizations, designed to carry out stand-alone programs, exhibited a reluctance to share confidential information [22]. This resulted in multiple organizations requesting information from grieving families who had already supplied it to another organization as well as lost time figuring out whether federal agencies or other levels of government had the best data for various purposes. On the contrary, some of the most successful activities during that recovery rested on years of relationship and trust building, including an unusual alliance between the media and emergency operations center which allowed for clear and quick communication on recovery to be coalesced and released through several media outlets [22]. Similarly, to alleviate anxiety in children and families impacted by the pandemics of severe acute respiratory syndrome (SARS) and H1N1 influenza, behavioral health professionals reported experiencing conflicts with public health, other behavioral health professionals, or other health care responses systems, inhibiting effective response [23]. In an evaluation conducted by the Center for Studying Health System Change, improvements in public health preparedness resulting from years of effort and sustained funding were due, in part, to establishing intersectoral collaborations and overcoming differences in organizational cultures and approaches to management among public health, fire, police, and emergency management agencies [24]. 

Partnerships between academic institutions and community-based organizations have had a demonstrable effect on enhancing community resilience. One study of an authentic academic-community partnership which provided experiential community health education in underserved communities in Arizona demonstrated an increase in community resilience by building human capital, increasing empowerment from community members working with students, increasing network connectivity by bringing a variety of professionals together, and increasing informational capital (i.e., generating relevant data for the community to support program development and grant applications) [25]. Evaluation of the program concluded that it may have been a catalyst for action and provided an organizational structure facilitating the development of new relationships and partnerships within the community, both key to resilience building [25,26]. 

While the majority of the current resilience literature pertains to health-related professions, a few articles highlighted the role of non-health and emergency preparedness workforce individuals in disaster preparedness and response. For a resilience-oriented workforce to flourish, organizational structures must be set up to integrate human and infrastructure systems sectors. Librarians and information specialists, such as those at the Disaster Information Management Research Center (DIMRC), can work with response personnel to meet their critical information needs [27]. Private lawyers, in addition to public attorneys, have a role to play to protect citizens when dealing with public health emergencies, particularly in the areas of liability insurance, sick leave, compensation policies, on-call requirements of health care professionals, and many others [28]. 

Further, Santos and colleagues have argued that in the context of a disaster, the workforce plays a dual role as both victims affected by the disaster and as a vital resource for recovery, and therefore, workforce recovery analysis in the context of disaster preparedness needs to be considered equally as important as critical infrastructure systems recovery [29]. The recognition of workforce criticality in disaster recovery, particularly in the healthcare, infrastructure, and education sectors, establishes a need for an integrated focus on the simultaneous recovery of the interdependent workforce, infrastructure, and regional economic systems. 

### 3.2. What Elements of Training Will Support Resilience Education?

The literature review identified several elements of training that could support resilience education. Several studies highlighted interprofessional education (IPE) programs [25,30]. The field of resilience is clearly transdisciplinary and requires practitioners to be trained in a wide variety of areas. IPE occurs when students from two or more professions learn about, from and with each other to enable effective collaboration; it has traditionally occurred in the health professions [31]. IPE grew out of a need for a strong, flexible, and collaborative workforce to confront highly complex challenges in healthcare. The field of resilience is now facing the same types of complexities. While resilience is a broad field, requiring skills from a wide variety of professions, much of the reviewed literature on training was much more narrow in scope, such as collaborations between schools of social work and public mental health departments [30] or incorporating disaster preparedness training into health education curricula [32,33], in particular, for nurses [34,35,36] and physicians [37,38]. IPE for the resilience-oriented workforce needs to be much more interdisciplinary than is currently in practice. For example, there is a need to integrate health sciences with urban planning and engineering education for resilience. Few models currently exist for this type of interdisciplinary educational experience. One that comes close is the framework for “Disaster Health” set forth by the World Association for Disaster and Emergency Medicine (WADEM) [39]. The consensus view in support of this framework resulted in a model which can facilitate the development of educational programs. The conceptual model includes the following components: primary disciplines (clinical and psychological, public health, and emergency and risk management); support disciplines (geography, engineering, anthropology); community response, resilience and communication; and socio-political context. This model may be a useful starting point for the type of IPE programs that can facilitate the development of a resilience-oriented workforce. A more focused needs assessment should be conducted to refine and validate this model. 

As discussed previously, community partnerships are a critical element for resilience [25]. Building and sustaining effective partnerships requires that the workforce is skilled in principles of community engagement. As has been recognized with other workforce groups (e.g., public health), professionals may not have the skill set necessary to work with local communities, and therefore, these competencies must be built into education and training programs [40]. A critical understanding of the unique roles and responsibilities of community organizations is also key [41]. 

Post-graduate and ongoing professional education is paramount for transdisciplinary fields. One study looked at how to enhance the environmental public health workforce, which parallels the resilience workforce in its broad and cross-cutting responsibilities. Particular challenges included a lack of ongoing training opportunities and the absence of a clearly defined career path [42]. To combat these challenges, ongoing professional education programs encompassing a full range of disciplines are needed to help resilience-oriented professionals expand their skill sets over time and adapt to the evolving needs of the community they serve. Training and professional education programs can be competency-based, which have proven effective in other fields, such as public health preparedness [43]. Competencies known to strengthen community resilience, such as engaging vulnerable populations in planning and increasing community self-sufficiency [6], should be targeted. Such competencies may include community outreach and engagement, decision making under uncertainty, holistic planning, and asset identification to understand what different sectors bring and build effective coalitions. Training on leadership, command structure, and communications is also needed [33].

Once a set of core competencies is established, mechanisms for assessment will need to be established. The disaster preparedness literature has described several ways in which this can be done. Several articles assessed health professionals’ skills and confidence against a set of competencies derived from government agency (e.g., Centers for Disease Control and Prevention) or national association publications [44]. Others discussed the role of different organizing bodies (education and professional organizations, accreditation and regulatory bodies, schools, and continuing education providers) and advanced curricula in preparing health professionals for disaster preparedness [45,46,47]. Skills assessment played out differently among different segments of the workforce. While a survey of medical students found that they were very willing to respond to a disaster scenario, education and training in disaster medicine in US medical schools was still deemed to be inadequate [48]. In a survey of emergency medical technicians (EMTs) and paramedics, a majority reported participating in disaster-preparedness training; however, it was less common for front-line emergency medical service professionals to have worked with other agencies and disciplines as part of the training [49]. In doctors, nurses, and allied health workers, individual perceived preparedness to respond to a disaster was correlated with prior training [50]. Finally, one study assessed the role of a volunteer workforce and found that volunteers in private organizations are willing to assist in disasters and have skills they can be used as part of disaster mitigation [51]. Further, volunteers have little expertise but a strong interest in being trained to contribute during disasters. 

### 3.3. What Are Leadership Models with a Resilience Orientation?

We did not find any existing leadership models with a resilience orientation. Instead we describe what resilience-oriented leadership looks like and elements that would be critical to include in a model, if developed. Wyche and colleagues assessed community resilience activities in workplace teams that became first responders for Hurricane Katrina survivors [52]. Teams with resilience-oriented behaviors were characterized by shared organizational identity, purpose, and values; mutual support and trust; role flexibility; active problem solving; self-reflections; shared leadership; and skill building. Freedom from administrative dominance and the existence of horizontal leadership (i.e., multiple skills and leaders without a need to assert dominance) was seen as critical. At the same time, community resilience requires individuals to commit to teamwork. The same study found that trust and cooperation amongst the team facilitated resilience in disaster response. Therefore, the right balance between workforce autonomy and operation as a collective entity must be struck in any resilience workforce leadership model. Because a resilience-oriented workforce must continuously balance the decisions and tradeoffs that come from facing both acute shocks and long-term stressors and marginalization, shared organizational identity and trust are crucial. Further, resilience-oriented leadership programs must support ways to navigate intersectoral, collaborative structures, create shared metrics and accountability frameworks, and articulate a cohesive plan that integrates shocks and stressors in daily practice. 

Further, appropriate leadership models can minimize problems associated with resource sharing. Lack of organizational familiarity has been cited as a barrier for effective partnerships between public health and emergency management agencies and local academic institutions [26]. However, by establishing leaders as points of contact between the organizations, and creating representation from academic institutions on planning bodies, as well as forming regional coordination centers, such barriers can be addressed.

## 4. Limitations to Existing Evidence

While much is still unknown about training to support a resilience-oriented education, it is clear that IPE and ongoing professional education will be critical in this developing field. The literature to date has identified a preliminary set of competencies that will be needed, as well as a starting point for a conceptual framework for educational programs for “disaster health”. But we also recognize that most of the articles focused on traditional health and healthcare professions, despite the recognition that resilience development will need disciplinary understanding across a range of human and infrastructure sectors. In a truly resilience-oriented framework, the full range of disciplines need to be considered as critical and not just as supporting the health disciplines and the orientation will need to move from preparedness, where many of the current lessons learned have emerged, to resilience. 

Once a conceptual framework for a resilience-oriented workforce is developed and validated it can be used to facilitate educational programs, including both graduate certificate programs (to build the capacity for new professionals from a wide variety of disciplines to be integrated, for example, through common symposia, seminars, and other networking opportunities) and continuing education programs. The framework can also be used to facilitate a “common language” for use by different members of the workforce [39], thus creating a shared sense of identity. The use of common terms and language across fields (e.g., healthcare, engineering, etc.) will be essential across the range of educational opportunities. Short courses can be developed to fill in gaps in knowledge, as identified from comparison with the framework. Ongoing professional development opportunities and a career advancement pathway that rewards cross-pollination activities to promote integration across disciplines are critical to building a strong and stable resilience-oriented workforce. Research is needed to develop models facilitating career progression and advancement that occurs in a more systems-oriented and integrated way. Few such models currently exist; however, one can look to universities and institutions that have developed sustainability curricula for guidance. These include the SDG Academy (https://courses.sdgacademy.org/), an initiative of the Sustainable Development Solutions Network, and the Stockholm Resilience Centre at Stockholm University (http://www.stockholmresilience.org/), which offers courses on “Systems Theory and Resilience Thinking” and “Governance and Management of Social-ecological Systems”.

Innovative partnerships will also be necessary to ensure that training takes a systems approach. For example, in New York City, the Workforce Field Building Hub (https://workforceprofessionals.org/workforce-field-building-hub/) serves as a central place for community-based organizations, for-profit service delivery agencies, government, education institutions, workforce philanthropies, organized labor, private sector employers, public libraries, employer-based training programs, and others to collectively respond to local and national trends and policy changes that impact workforce development. The Workforce Field Building Hub is meant to be a systems-building initiative that brings together key leaders from across the interdisciplinary and diverse local and national workforce community to identify common issues and solutions to strengthen the workforce ecosystem. A similar type of approach could help to develop and sustain the new resilience-oriented workforce.

There is little empirical data to inform structures and leadership models for a resilience-oriented workforce. However, from the disaster recovery literature, we recognize the importance of taking a systems approach and breaking down silos between sectors and types of stakeholders. Such an approach relies on structures and operational frameworks that facilitate inter-departmental and intersectoral data and resource sharing. Areas that cannot be overlooked include how leaders will develop collaborations across sectors, such as through memorandums of understanding and other contractual arrangements to facilitate such structures; how to create joint incentives across sectors; and how to optimally work through entrenched politics among disciplinary factions. Further, traditional approaches of top-down management may be less effective than horizontal leadership styles, where individuals are empowered in decision-making. Research is needed to understand how to align such an approach with creating a shared sense of identity within the resilience-oriented workforce.

Of note, we found limited articles with a focus on the integration of economic, infrastructure, and human resilience. While we did not hand search bibliographies for this literature review, our results indicate that further research is needed to build an evidence base to support the development of an integrated resilience-oriented workforce.

## 5. Conclusions

In summary, while deepening the field of resilience will require a trained and capable resilience-oriented workforce, few studies in the peer-reviewed literature provide data to support what that workforce looks like and how it is cultivated. Future research will need to collect and synthesize data to support metrics to evaluate the development of a resilience-oriented workforce. In addition, since creating a resilient community is a shared responsibility across many actors and organizations, future research will need to examine how effectively a resilience-oriented workforce engages and empowers individuals and organizations across a community. Table 2 points to this and other recommendations to move the workforce forward.

We identified that a resilience-oriented workforce spans many disciplines and requires a collaborative model that promotes information sharing structures among organizations, between organizations and individuals, across multiple levels and branches of government, and the private sector (e.g., through coalitions). IPE programs, expertise in community partnership and engagement, and dedicated post-graduate and professional development training are all needed to build a resilience-oriented workforce. Capability-based assessment tools are also needed to help track and monitor how leaders begin to embrace resilience systems thinking and to allow for career advancement that accounts for this integration. Leadership models should foster a balance between workforce autonomy and operation as a collective entity. Further, while the literature reviewed for this article were heavily focused in traditional health venues, a workforce that appreciates resilience thinking, will need to become better versed in the intersection of human systems and infrastructure systems integration.

## Figures and Tables

**Table 1:** Resilience-oriented workforce studies.

Author	Year	Discipline	Workforce Segment 1	Workforce Segment 2	Type of Study	Research Question(s)	Summary
Dawes et al.	2004	Disaster Preparedness/Response	Emergency Response	Responders	Case Study	Organization	Summarizes human and infrastructure issues post disaster: importance of data quality, usability and resource sharing among responders; discusses information policy challenges for workforce response
desVignes-Kendrick et al.	2005	Disaster Preparedness/Response	Other	Lawyers	Session abstract	Organization	Discusses the role of private lawyers in dealing with public health emergencies.
Katz et al.	2006	Disaster Preparedness/Response	Health	LHD executives, community partners, hospital executives, community health center executives	Longitudinal evaluation	Organization	Collaborative relationships developed for bioterrorism preparedness have proved useful in addressing other threats. Major ongoing challenges include funding constraints, inadequate surge capacity, public health workforce shortages, competing priorities, and jurisdictional issues.
Phillips	2013	Disaster Preparedness/Response	Other	Librarians	Review	Organization	Describes the Disaster Information Management Research Center (DIMRC) develops and provides access to health information resources and technology for disaster preparedness, response, and recovery.
Santos et al.	2014	Interdependent workforce, infrastructure, and economic systems	Other	general workforce	Review	Organization	Highlights importance of workforce sectors in formulating synergistic preparedness and recovery policies for interdependent infrastructure and regional economic systems.
Sprang and Silman	2015	Disaster Preparedness/Response	Health	Behavioral health professionals	Review/Commentary	Organization; Training	Describes five principles to integrate behavioral health services in the public health disaster response plan which use a strengths-based approach to prioritize resilience; underscore the importance of context, collaboration, and coordination; recognize the unique needs of pediatric populations; and guide ongoing training and content development in the area of biopsychosocial responses to health-related disasters.
Dunlop et al.	2016	Disaster Preparedness/Response	Health	Public health and emergency management workers, academics	Needs Assessment/Survey/Focus groups	Organization; Leadership	Explored the opinions of leaders of public health and emergency management agencies and academic institutions regarding the facilitators for and barriers to effective collaboration for disaster preparedness and response. Recognized barriers to engagement included unfamiliarity of organizational personnel, concerns about ownership of outputs resulting from the collaboration, and differences in organizational culture and modus operandi. On-going relationships through shared training of students and staff and participation in community-level partner meetings facilitated collaboration in disaster response as does having a recognizable point of contact that can comprehensively represent academic institutional resources. Legal issues were identified as both facilitators and barriers to engagement.
Barnett et al.	2005	Disaster Preparedness/Response	Health	LHD Workers	Presentation of Workforce Training Program	Training	Describes a competency-based training for public health emergency response.
Stanley	2005	Disaster Preparedness/Response	Health	Nurses	Review/Commentary	Training	Roles of key entities are essential for education’s successful implementation of disaster preparedness: education and professional organizations, accreditation and regulatory bodies, schools of nursing, and continuing education providers.
Davies and Moran	2005	Disaster Preparedness/Response	Health	Nurses	Review/Commentary	Training	Describes role of nursing workforce in disaster preparedness
Mosca et al.	2005	Disaster Preparedness/Response	Health	Nurses (school)	Needs Assessment	Training	Assessed bioterrorism and disaster preparedness needs of school nurses assessed; low confidence in preparedness capabilities across almost all categories was reported; high training need was identified across almost all competencies.
Veenema	2006	Disaster Preparedness/Response	Health	Nurses	Presentation of Workforce Training Program	Training	Workforce development and nurse preparedness for schools of nursing.
Archer and Seynaeve	2007	Disaster Preparedness/Response	Health	Health professionals	Issues Paper/Framework	Training	World Association for Disaster and Emergency Medicine (WADEM) meeting convened in support of a framework for “Disaster Health”, which included: (1) primary disciplines; (2) support disciplines; (3) community response, resilience, and communication; and (4) socio-political context.
Douglas	2007	Disaster Preparedness/Response	Health	Nurses and Paramedics	Presentation of Workforce Training Program	Training	Describes multi-agency collaborative approach to develop modules on the management of mass casualty events and incidents involving hazardous substances, offered to registered nurses and registered paramedics.
Fulmer et al.	2007	Disaster Preparedness/Response	Other	University volunteers	Survey on volunteer willingness for disaster response	Training	Survey results suggest that volunteers can and will help and that disaster preparedness drills are a logical next step for university-based volunteers.
Resnick et al.	2007	Environmental public health	Health	LHD Workers	Review	Training	Obstacles for strengthening the environmental public health workforce include recruitment shortfalls, inability to retain qualified staff, impending retirements, inadequate training opportunities, insufficient compensation, and the absence of a robust career advancement pathway.
Black et al.	2008	Mental health resiliency	Health	Social work and public mental health students	Presentation of IPE program	Training	Describes an innovative statewide collaboration between schools of social work and public mental health departments to transform social work curriculum and address the workforce crisis in public mental health service system.
Kaiser et al.	2009	Disaster Preparedness/Response	Health	Medical students	Survey/Needs Assessment	Training	Survey results indicate future physicians’ willingness to respond to disasters, but education and training in disaster medicine and public health preparedness offered in US medical schools is inadequate.
Morrison and Catanzaro	2010	Disaster Preparedness/Response	Health	Nursing students	Presentation and evaluation of training simulation exercise	Training	Describes a public health emergency simulation exercise with undergraduate senior nursing students enrolled in a public health clinical course.
Potter et al.	2010	Disaster Preparedness/Response	Health	Public health workers	Review	Training	Reviews of progress in preparedness training for the public health workforce should be repeated in the future. Governmental investment in training for preparedness should continue. Future training programs should be grounded in policy and practice needs, and evaluations should be based on performance improvement.
Catlett et al.	2011	Disaster Preparedness/Response	Health	EMS Physicians	Resource document	Training	Advocates for a strong emergency medical services (EMS) role in all phases of disaster management—preparedness, response, and recovery.
Fernandez et al.	2011	Disaster Preparedness/Response	Health	EMTs and Paramedics	Needs Assessment	Training	A majority of nationally certified EMT-Basics and paramedics reported participating in both individual and multiagency disaster-preparedness training. A large majority of respondents reported feeling adequately prepared to respond to man-made and natural disasters and the perception of preparedness correlated with hours of training. Some areas for improvement were identified.
Slack et al.	2013	Community resilience	Health	Health Science students	Evaluation of IPE program	Training	By acting as a catalyst, a community based interprofessional program can affect components of community resilience/capacity, primarily human, social, and informational capital.
Lim et al.	2013	Disaster Preparedness/Response	Health	Health care workers (Physicians, Nurses, Allied Health workers)	Survey/Needs Assessment	Training	Survey indicates that health care workers fare poorly in their perception of their individual preparedness. Identifies Important factors that might contribute to improving this perception at the individual and institution level.
Baack and Alfred	2013	Disaster Preparedness/Response	Health	Nurses (rural)	Survey/Needs Assessment	Training	Most rural nurses are not confident in their abilities to respond to major disaster events. The nurses who were confident were more likely to have had actual prior experience in disasters or shelters.
Kumar and Weibley	2013	Disaster Preparedness/Response	Health	Physicians	Review/Commentary	Training	Describes physicians’ obligations, role, education, preparation, and response for disasters.
Veenema et al.	2016	Disaster Preparedness/Response	Health	Nurses	Needs Assessment/SME interviews	Training	Describes a vision for the future of disaster nursing, and identifies current barriers and opportunities to advance professional disaster nursing. Includes recommendations for nursing practice, education, policy, and research, as well as implementation challenges.
Achora and Kamanyire	2016	Disaster Preparedness/Response	Health	Nurses	Review/Commentary	Training	Highlights the current state of nursing education and training in disaster management, both generally and in Oman.
Wyche et al.	2011	Disaster Preparedness/Response	Emergency Response	First responders	Evaluation of work place teams-survey, focus groups, interviews	Leadership	Community resilience activities were assessed in workplace teams that became first responders for Hurricane Katrina survivors. Resilient behaviors were characterized by: shared organizational identity, purpose, and values; mutual support and trust; role flexibility; active problem solving; self-reflection; shared leadership; and skill building.

**Table 2:** Recommendations to move the resilience-oriented workforce forward.

Category	Recommendation
Improve the evidence base	Develop a core set of metrics for workforce evaluation.
	Conduct research to determine what does and does not work (e.g., training, organizational structures, leadership models) for achieving pre-defined metrics.
Develop Competencies/Training	Identify, validate, and assess a set of competencies that support a resilience-oriented workforce.
	Develop resilience-oriented interprofessional education programs at the graduate and continuing education levels.
	Incorporate community-based partnerships into training programs to develp skills to work with local communities.
Faciltate Organizational Structures	Use common language across fields.
	Develop integrated organizational frameworks for doing business across human and infrastructure systems.
Cultivate Leadership Models	Determine incentive structures that promote horizontal leaderships and shared identity.
	Create career advancement opportunities that recognize interdisciplinary and intersectoral experience.
	Establish mechanisms of collaboration across sectors and disciplines (e.g., through regional coordination centers).

## References

[1] Department of Homeland Security Disaster Declarations. www.fema.gov/disasters.

[2] Keeley B. (2015). Income Inequality: The Gap between Rich and Poor.

[3] NOAA National Centers for Environmental Information (NCEI) (2016). U.S. Billion-Dollar Weather and Climate Disasters. https://www.ncdc.noaa.gov/billions/.

[4] Blair J.P., Schweit K.W. (2014). A Study of Active Shooter Incidents in the United States between 2000 and 2013.

[5] Abramson D.M., Grattan L.M., Mayer B., Colten C.E., Arosemena F.A., Bedimo-Rung A., Lichtveld M. (2015). The resilience activation framework: A conceptual model of how access to social resources promotes adaptation and rapid recovery in post-disaster settings. J. Behav. Health Serv. Res..

[6] Chandra A., Acosta J., Howard S., Uscher-Pines L., Williams M., Yeung D., Garnett J., Meredith L.S. (2011). Building community resilience to disasters: A way forward to enhance national health security. Rand Health Q..

[7] Chandra A., Williams M., Plough A., Stayton A., Wells K.B., Horta M., Tang J. (2013). Getting actionable about community resilience: The Los Angeles county community disaster resilience project. Am. J. Public Health.

[8] Acosta J., Chandra A., Madrigano J. (2017). An Agenda to Advance Integrative Resilience Research and Practice.

[9] U.S. Department of Homeland Security (2011). National Disaster Recovery Framework.

[10] US Department of Health and Human Services (2009). National Health Security Strategy of the United States of America.

[11] Centers for Disease Control and Prevention (2011). Public Health Preparedness Capabilities: National Standards for State and Local Planning.

[12] United States Department of Housing and Urban Development National Disaster Resilience Competition. http://portal.hud.gov/hudportal/documents/huddoc?id=NDRCFactSheetFINAL.pdf.

[13] Rockefeller Foundation 100 Resilient Cities. http://www.100resilientcities.org.

[14] Ottoson J.M., Green L.W., Beery W.L., Senter S.K., Cahill C.L., Pearson D.C., Greenwald H.P., Hamre R., Leviton L. (2009). Policy-contribution assessment and field-building analysis of the robert wood johnson foundation’s active living research program. Am. J. Prev. Med..

[15] Coaffee J. (2013). Towards next-generation urban resilience in planning practice: From securitization to integrated place making. Plan. Pract. Res..

[16] Graham H. (2002). Building an inter-disciplinary science of health inequalities: The example of lifecourse research. Soc. Sci. Med..

[17] Harriss J. (2002). The case for cross-disciplinary approaches in international development. World Dev..

[18] Ernstson H., van der Leeuw S.E., Redman C.L., Meffert D.J., Davis G., Alfsen C., Elmqvist T. (2010). Urban transitions: On urban resilience and human-dominated ecosystems. AMBIO J. Hum. Environ..

[19] Harvard Transdisciplinary Research in Energetics and Cancer Center. https://www.hsph.harvard.edu/trec/about-us/definitions/.

[20] Kennedy V.C., Moore F.I. (2001). A systems approach to public health workforce development. J. Public Health Manag. Pract..

[21] Handler A., Issel M., Turnock B. (2001). A conceptual framework to measure performance of the public health system. Am. J. Public Health.

[22] Dawes S.S., Cresswell A.M., Cahan B.B. (2004). Learning from crisis. Soc. Sci. Comput. Rev..

[23] Sprang G., Silman M. (2015). Using professional organizations to prepare the behavioral health workforce to respond to the needs of pediatric populations impacted by health-related disasters: Guiding principles and challenges. Disaster Med. Public Health Prep..

[24] Katz A., Staiti A.B., McKenzie K.B. (2006). Preparing for the unknown, responding to the known: Communities and public health preparedness. Health Aff..

[25] Slack M.K., McEwen M.M. (2013). Perceived impact of an interprofessional education program on community resilience: An exploratory study. J. Interprof. Care.

[26] Dunlop A.L., Logue K.M., Vaidyanathan L., Isakov A.P. (2016). Facilitators and barriers for effective academic-community collaboration for disaster preparedness and response. J. Public Health Manag. Pract..

[27] Phillips S.J. (2013). The national library of medicine’s disaster information management research center. Front. Public Health.

[28] DesVignes-Kendrick M., Matthews G.W., Steeg S.K., Zinder S.F. (2005). The private bar: A force for public health. J. Law Med. Ethics.

[29] Santos J.R., Herrera L.C., Yu K.D., Pagsuyoin S.A., Tan R.R. (2014). State of the art in risk analysis of workforce criticality influencing disaster preparedness for interdependent systems. Risk Anal. Off. Publ. Soc. Risk Anal..

[30] Black J., Morris T., Harbert A., Mathias C. (2008). Educational collaboration in psychiatric disability, rehabilitation, and recovery: Developing transformative solutions. J. Soc. Work Disabil. Rehabil..

[31] World Health Organization (2010). Framework for Action on Interprofessional Education & Collaborative Practice.

[32] Veenema T.G. (2006). Expanding educational opportunities in disaster response and emergency preparedness for nurses. Nurs. Educ. Perspect..

[33] Potter M.A., Miner K.R., Barnett D.J., Cadigan R., Lloyd L., Olson D.K., Parker C., Savoia E., Shoaf K. (2010). The evidence base for effectiveness of preparedness training: A retrospective analysis. Public Health Rep..

[34] Morrison A.M., Catanzaro A.M. (2010). High-fidelity simulation and emergency preparedness. Public Health Nurs..

[35] Achora S., Kamanyire J.K. (2016). Disaster preparedness: Need for inclusion in undergraduate nursing education. Sultan Qaboos Univ. Med. J..

[36] Veenema T.G., Griffin A., Gable A.R., MacIntyre L., Simons R.N., Couig M.P., Walsh J.J., Lavin R.P., Dobalian A., Larson E. (2016). Nurses as leaders in disaster preparedness and response—A call to action. J. Nurs. Scholarsh..

[37] Catlett C.L., Jenkins J.L., Millin M.G. (2011). Role of emergency medical services in disaster response: Resource document for the national association of ems physicians position statement. Prehosp. Emerg. Care.

[38] Kumar A., Weibley E. (2013). Disaster management and physician preparedness. South. Med. J..

[39] Archer F., Seynaeve G. (2007). International guidelines and standards for education and training to reduce the consequences of events that may threaten the health status of a community. A report of an open international wadem meeting, Brussels, Belgium, 29–31 October 2004. Prehosp. Disaster Med..

[40] Amodeo A.R. (2003). Commentary: Developing and retaining a public health workforce for the 21st century: Readiness for a paradigm shift to community-based public health. J. Public Health Manag. Pract..

[41] Acosta J., Chandra A. (2013). Harnessing a community for sustainable disaster response and recovery: An operational model for integrating nongovernmental organizations. Disaster Med. Public Health Prep..

[42] Resnick B., Zablotsky J., Farrow O., Glotfelty R., Heard P., Kelly S., Mitchell C., Phillips F., Burke T. (2007). Enhancing the maryland environmental public health workforce: A collaborative approach. J. Environ. Health.

[43] Barnett D.J., Everly G.S., Parker C.L., Links J.M. (2005). Applying educational gaming to public health workforce emergency preparedness. Am. J. Prev. Med..

[44] Mosca N.W., Sweeney P.M., Hazy J.M., Brenner P. (2005). Assessing bioterrorism and disaster preparedness training needs for school nurses. J. Public Health Manag. Pract..

[45] Stanley J.M. (2005). Disaster competency development and integration in nursing education. Nurs. Clin. N. Am..

[46] Davies K., Moran L. (2005). Nurses need advanced skills in disaster health care. Br. J. Nurs..

[47] Douglas V. (2007). Developing disaster management modules: A collaborative approach. Br. J. Nurs..

[48] Kaiser H.E., Barnett D.J., Hsu E.B., Kirsch T.D., James J.J., Subbarao I. (2009). Perspectives of future physicians on disaster medicine and public health preparedness: Challenges of building a capable and sustainable auxiliary medical workforce. Disaster Med. Public Health Prep..

[49] Fernandez A.R., Studnek J.R., Margolis G.S., Mac Crawford J., Bentley M.A., Marcozzi D. (2011). Disaster preparedness of nationally certified emergency medical services professionals. Acad. Emerg. Med..

[50] Lim G.H., Lim B.L., Vasu A. (2013). Survey of factors affecting health care workers’ perception towards institutional and individual disaster preparedness. Prehosp. Disaster Med..

[51] Fulmer T., Portelli I., Foltin G.L., Zimmerman R., Chachkes E., Goldfrank L.R. (2007). Organization-based incident management: Developing a disaster volunteer role on a university campus. Disaster Manag. Response.

[52] Wyche K.F., Pfefferbaum R.L., Pfefferbaum B., Norris F.H., Wisnieski D., Younger H. (2011). Exploring community resilience in workforce communities of first responders serving katrina survivors. Am. J. Orthopsychiatry.

